# The Crosstalk between Ovarian Cancer Stem Cell Niche and the Tumor Microenvironment

**DOI:** 10.1155/2017/5263974

**Published:** 2017-07-27

**Authors:** Manuel Varas-Godoy, Gregory Rice, Sebastián E. Illanes

**Affiliations:** ^1^Laboratory of Reproductive Biology, Center for Biomedical Research, Faculty of Medicine, Universidad de Los Andes, Santiago, Chile; ^2^Department of Obstetrics and Gynaecology, Faculty of Medicine, Universidad de Los Andes, Santiago, Chile; ^3^Centre for Clinical Diagnostics, Royal Brisbane and Women's Hospital, University of Queensland Centre for Clinical Research, Brisbane, QLD, Australia; ^4^Department of Obstetrics and Gynaecology, Clínica Davila, Santiago, Chile

## Abstract

Ovarian cancer is one of the most important causes of cancer-related death among women in the world. Despite advances in ovarian cancer treatment, 70–80% of women who initially respond to therapy eventually relapse and die. There is evidence that a small population of cells within the tumors called cancer stem cells (CSCs) could be responsible for treatment failure due to their enhanced chemoresistance and tumorigenicity. These cells reside in a niche that maintains the principal properties of CSCs. These properties are associated with the capacity of CSCs to interact with different cells of the tumor microenvironment including mesenchymal stem cells, endothelial cells, immune cells, and fibroblasts, promoting cancer progression. This interaction can be mediated by cytokines, growth factors, lipids, and/or extracellular vesicles released in the CSC niche. In this review, we will discuss how the interaction between ovarian CSCs and the tumor microenvironment can contribute to the maintenance of the CSC niche and consequently to tumor progression in ovarian cancer.

## 1. Introduction

Among the different gynecological cancer, ovarian cancer (OVCA) is the most lethal one in women worldwide. Although OVCA accounts for only 3% of all cancer incidents, 6% of cancer-related deaths are caused by ovarian cancer, making it the fifth leading cause of cancer mortality in women [1]. The main contributing factor to the high mortality rate of OVCA is late diagnosis, and although the use of first-line chemotherapy (e.g., paclitaxel-platinum combination) is initially effective for most patients, around 70% of women with advanced OVCA (stages 3-4) relapse within a few years after treatment and die due to the development of drug resistance [2, 3]. A small population of cells termed cancer stem cells (CSCs) has been identified as important contributors to drug resistance in ovarian cancer because they possess molecular and cellular mechanisms identified as important contributors of chemoresistance [4–7].

CSCs constitute a subset of cells with self-renewal and differentiation properties that are distinguished from the bulk of tumor cells by their exclusive ability to perpetuate the growth of a malignant cell population indefinitely [8]. CSCs have different cellular characteristics involved in cancer pathogenesis, such as tumorigenesis, metastasis, and tumor resistance [8, 9]. The presence of CSCs, therefore, offers a plausible explanation for the high rate of relapse, even some months after the therapy, with an initial successful treatment [3, 10, 11]. The explanation for relapse has usually been explained by tumor cells acquiring a resistant phenotype; however, studies have shown that resistance can be associated with the capacity of CSCs to resist the initial treatment and then to interact with different cell types of the tumor microenvironment to promote relapse and cancer progression [12, 13].

## 2. Tumor Microenvironment

The tumor microenvironment is the combination of noncancerous cells and the proteins produced by all the cells present in the tumor. The group of noncancer cells in the tumor is also defined as stroma and is composed of endothelial cells, cancer-associated fibroblasts (CAFs), adipocytes, mesenchymal cells, mesenchymal stem cells (MSCs; bone marrow derived (BM-MSCs) or carcinoma associated (CA-MSCs)), and cells from the immune and inflammatory systems (tumor-associated macrophages (TAM), regulatory T cells, etc.) [14, 15]. The participation of tumor stroma components in carcinogenesis and how the different cells of the tumor microenvironment contribute to induce tumor progression and metastasis has been extensively described [16, 17]. Stromal cells could be implicated in the acquisition of a specific phenotype by different processes, such as cell-cell and cell-matrix interaction, local release of soluble factors, generation of specific niches within the tumor, or conversion of cancer cells to CSCs [14, 15]. In the case of OVCA, the importance of the microenvironment in tumor progression can be explained by the bidirectional interaction between OVCA cells and their own stroma modulating the contents of the ascitic fluid promoting the protumoral phenotype of the stromal cells and regulating processes to favor tumor progression [18–22]. A good example of this interaction is tumor vascularization, which is essential for tumor growth and survival. The vascular endothelial growth factor (VEGF) is the most potent proangiogenic factor and is secreted by different types of cells including MSCs and endothelial and tumor cells [23]. In OVCA, VEGF induces the expression of CXCL12 receptor in vascular endothelial cells (VECs) and the hypoxic condition of the tumor induces the secretion of CXCL12 and VEGF acting together to induce angiogenesis [24]. MSCs have also been implicated in promoting angiogenesis by induction of VEGF and HIF1*α* expression in ovarian cancer [19]. In addition, OVCA cells secrete lysophosphatidic acid (LPA), a potent bioactive lysophospholipid that activates the expression and secretion of CXCL12 by MSCs, enhancing the resistance of OVCA cells to hyperthermia [25]. Several studies show the role of MSCs in tumor progression and how these cells interact with OVCA cells in response to different stimulus [19, 26, 27].

Cells acquiring tumorigenic traits (i.e., unregulated cell proliferation and resistance to cell death) are insufficient for tumor progression, and for that reason, multiple cell types are involved in this process, requiring effective cell-to-cell communication between cancer cells and local/distant microenvironments [28]. Cytokines, growth factors, and extracellular vesicles (EVs), including exosomes, could play an important role in this interaction and can influence proliferation, angiogenesis, chemoresistance, and metastasis [18]. In the next sections, we will discuss the importance of all these factors in the maintenance of the CSC niche and tumor progression.

## 3. Ovarian CSCs and Inflammatory Network

One of the hallmarks in cancer is the effect of inflammation in the tumor microenvironment and how the different components involved in the inflammatory process can contribute to tumor development [28, 29]. Several studies have shown the importance of different cytokines secreted by the tumor microenvironment in the regulation of CSCs [30]. In the case of ovarian cancer, it is known that an inflammatory state is considered a risk factor and can be associated with ovarian cancer development, drug resistance, and metastasis [31, 32]. Several cytokines have been described in circulation, ascites, and cyst fluid of patients with ovarian cancer [33–38] and also in the stroma and epithelium of tumors [39]. The presence of cytokines in the tumor stroma raises the possibility of activating signaling pathways related to the inflammatory network in all cell types of the stroma, including the ovarian CSCs. One of the cytokines present in the tumor microenvironment of ovarian cancer is IL-17 [40]. Xiang and collaborators demonstrated that IL-17 in ovarian cancer is produced by CD4+ T cells and CD68+ macrophages, tumor-associated macrophages (TAM) in the ovarian CSC niche, and the IL-17 receptor is expressed in a population of CD133+ CSCs [41]. The activation of this signaling pathway promotes self-renewal of the ovarian CSCs mediated by nuclear factor NF*κ*B and p38 mitogen-activated protein kinase (MAPK) signaling pathway, contributing to the ovarian cancer progression [41].

Among the cell types associated with the tumor microenvironment, M2 macrophages, a type of TAM, have a significant effect on tumor progression in several types of cancer, including ovarian cancer. These cells can secrete different factors, including VEGF, TGF-*β*, PPAR-*γ*, IL-10, and IL-17 [42–45]. As we mentioned before, CD68+ macrophages can induce the self-renewal of ovarian CSCs, but evidence shows that ovarian CSCs can induce polarization of M2 macrophages. Transwell assays of ovarian CSCs with monocytes showed an increase in monocyte differentiation to macrophages with M2 phenotype, increase in the IL-10, decrease in TNF-*α*, and activation of PPAR-*γ* and NF*κ*B [46, 47]. These results indicate that soluble factors, including cytokines, secreted by the ovarian CSCs, contribute to the M2 macrophage polarization to support the self-renewal of themselves.

In ovarian cancer, the proinflammatory state is not only induced by cells of the immune system. Other cells of the tumor microenvironment can contribute to generate different cytokines affecting the ovarian CSC niche. For example, in OVCA, metastasis occurs commonly in the omentum by the overexpression of ErbB3 in the tumor cells and the overexpression of neuregulin 1 in the omentum [48]. The omentum is an organ primarily composed of adipocytes, and these cells also can promote homing, migration, and invasion of ovarian cancer cells through release of cytokines such as IL-8 and IL-6 [49]. Both cytokines are also released by other sources in the tumor microenvironment and have been shown to regulate CSCs in other types of tumor [50–53]. Cytokines secreted by adipose tissue in ovarian cancer, therefore, may also regulate mechanisms related to CSCs. For example, one mechanism used for ovarian CSCs to acquire chemoresistance is mediated by the high expression of Bcl_xl_, and IL-6 secreted by the adipocyte increases the levels of Bcl_xl_ in chemosensitive ovarian cancer cells using the same mechanism used by ovarian CSC enhancing the proliferation, sphere formation, and tumorigenesis of ovarian cancer cells [54]. This data supports a role for cytokines released by adipocytes in ovarian cancer that can regulate CSCs.

As described before, in the tumor microenvironment, there are several types of interactions between different cell types. One of them is autocrine interaction that includes CSCs, which are able to secrete cytokines that will activate inflammatory signaling pathways in the same cell. For example, Wang and collaborators reported that CD133+ ovarian CSCs have the IL-23/IL-23 receptor axis activated, and the activation of this pathway promotes self-renewal and formation of ovarian CSCs by activation of the signaling pathways STAT3 and NF*κ*B, thus contributing to tumor progression [55]. In the same way, CD133+ cells have CCL5 and its receptors upregulated, and their autocrine activation promotes invasion and migration via NF*κ*B-mediated MMP-9 upregulation [56]. On the other hand, stem cells have the capacity to differentiate into other cell types by expressing different phenotypes, and CSCs are no exception. There is evidence that ovarian CSCs are able to differentiate into stromal cells supporting tumoral processes. One of these processes is angiogenesis, where new blood vessels are required for solid tumor maintenance, progression, and metastasis [57]. Alvero and colleagues demonstrated that CD44+ ovarian cancer cells, another subpopulation of ovarian cancer cells with stem-like properties different from CD133+ ovarian cancer cells, have the capability to be differentiated into a CD44+/VE-cadherin+/CD34+ cells phenotype and mimic the behavior of normal endothelial cells forming vessel-like structures in a VEGF-independent manner [58]. Supporting this discovery, Tang and colleagues showed that ovarian CSCs can activate NF*κ*B and STAT3 signaling secreting CCL5 and activating this pathway in an autocrine manner to allow its own differentiation into endothelial cells to improve tumor angiogenesis [59]. These data highlights the importance of the inflammatory network in the tumor microenvironment, as well as the mechanisms by which cytokines can support the ovarian CSC niche.

## 4. Ovarian CSCs and Growth Factors

Growth factors play an important role in maintaining tissue homeostasis under physiological conditions, but in cancer, the same growth factors can be involved in tumor progression [60]. As with cytokines, cells from the tumor microenvironment are able to secrete growth factors and regulate processes that are important for tumor development such as angiogenesis and metastasis, including the function of CSC and tumor initiation [61, 62].

Cancer-associated fibroblasts (CAFs) are key components of the tumor stroma and could have an important role in ovarian cancer progression and metastasis [63]. Histological examination and gene expression analysis of ovarian tumor tissues have shown abundant fibrous stroma formation, overexpression of fibroblast growth factor 4 (FGF4), and stem cell-associated genes in samples enriched with CSCs in the presence of fibroblasts [64]. *In vivo* studies demonstrated that the capability to generate ovarian CSCs was enhanced in the presence of CAFs and the capacity of the fibroblast to enhance CSC properties was suppressed by knockdown of the FGF4 receptor (FGFR2), expressed preferentially in ovarian CSCs [64]. Supporting these data, there is evidence that the activation of FGF signaling can control the expansion and self-renewal of CSCs [65–67]. Moreover, FGF is able to induce angiogenesis through the autocrine induction of VEGF secretion [68]. VEGF is the master regulator of angiogenesis [69], but the function of VEGF in cancer is not limited to the generation of new blood vessels; it can also promote CSC properties in certain cancers [70]. In ovarian cancer, VEGF-A, a member of the VEGF family, stimulates ovarian CSCs through VEGFR2-dependent Src activation to upregulate the stem cell factor B cell-specific Moloney murine leukemia virus integration site 1 (Bmi1) [71].

CAFs can also participate in the generation and maintenance of the CSC niche via activation of the insulin growth factor receptor (IGF-IR), inducing Nanog expression and stem cell phenotype in cancer cells [72]. In ovarian cancer, the IGF signaling is involved in tumor progression and chemoresistance [73] and the activation of IGF-1R-AKT signaling by different chemotherapeutics agents increase the expression of genes involved in self-renewal (*Oct4/Sox2/Nanog*) and imparts functional heterogeneity in the ovarian CSCs during acquirement of chemoresistance [74].

Evidence also suggests that MSCs are recruited to the tumor microenvironment. A special type of MSCs has been identified associated to ovarian carcinoma called carcinoma-associated MSCs (CA-MSCs) and is present in the majority of human ovarian tumor samples [75]. One of the characteristic of the CA-MSCs is the upregulation of the TGF-*β* superfamily/BMP family members in comparison with control MSCs, and this activation in the BMP signaling pathways increases the population of ovarian CSCs promoting the CSC proliferation [75]. Other factors such as TNF-*α* and TGF-*α*, released by different types of cells in the tumor microenvironment, have also been identified with a potential role in the ovarian cancer progression [62]. The exact nature of their interactions with CSCs remains to be clearly established.

## 5. Ovarian CSCs, MicroRNA Regulation, and Extracellular Vesicles

Different cell types, including CSCs, can regulate the expression of small noncoding RNAs called microRNAs (miRNAs) to regulate several processes [76, 77]. In order to maintain the stemness of cancer cells, the tumor microenvironment can modulate the expression of miRNAs. Cui and collaborators showed that myeloid-derived suppressor cells (MDSCs), components of the tumor microenvironment, stimulate the expression of miRNA-101 in ovarian cancer cells and subsequently repress the corepressor gene C-terminal binding protein-2 (CtBP2), resulting in an increase in cancer cell stemness and metastatic and tumorigenic potential [78]. How the MDSCs regulate the expression of this miRNA is still unclear, but one of the possibilities is that MDSCs could transfer these miRNAs by extracellular vesicles (EVs). EVs are small membrane vesicles capable of transferring contents between cells to function in cell-cell communication [79].

In the last decades, the communication and exchange of proteins, mRNA, and miRNAs mediated by EVs within the tumor microenvironment has acquired a big relevance in the regulation of tumor processes such as metastasis and chemoresistance [79–81], all processes where CSCs are involved. Some studies have described the role of EVs derived from CSCs in the tumor progression of renal, prostate, and breast cancer [82–84]. In ovarian cancer, although the release of EVs is very important to mediate tumor progression [85–87], the interaction between the tumor microenvironment and the ovarian CSCs mediated by EVs is still unclear.

## 6. Conclusion

The available evidence supports the hypothesis that the niche of ovarian CSCs plays an important role in the initiation of the tumor, but this role would not be possible without the interaction of the niche with the tumor microenvironment. This interaction, mediated by different types of factors, can be considered bidirectional; this communication allows the ovarian CSCs to maintain the stemness of the niche while differentiating the CSCs to other cell types of the tumor microenvironment in order to support tumor progression. Similarly, CSCs may modulate the function of different cells in the tumor microenvironment to support these tumorigenic properties.

**Figure 1:**
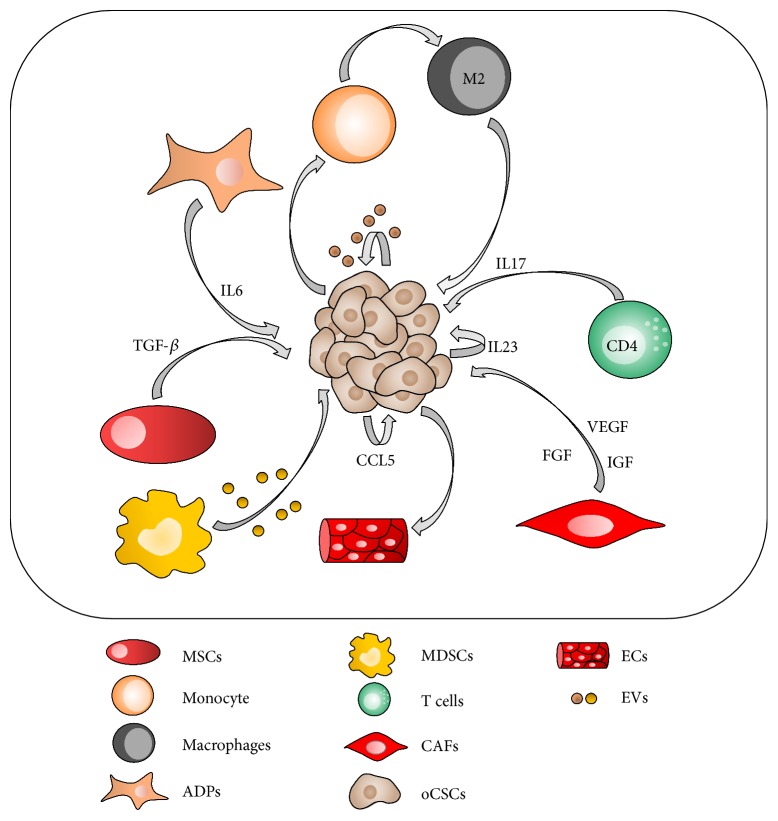
Schematic representation of the interaction between the ovarian cancer stem cell niche and the tumor microenvironment. T cells and M2 macrophages mediate self-renewal of oCSCs by secretion of IL-17. ADPs support tumorigenesis of oCSCs by secretion of IL-6. CAFs mediate self-renewal of oCSCs by secretion of FGF, VEGF, and IGF. MSCs mediate tumorigenesis of oCSCs by secretion of TGF-*β*. oCSCs induce differentiation of monocyte to M2 macrophages. oCSCs (CD133+) induce its own self-renewal by autocrine activation of IL-23 secretion. oCSCs induce tumorigenesis by CCL5 secretion (CD133+) and EV secretion. oCSCs (CD44+) induce its own differentiation to ECs by secretion of CCL5. MSCs: mesenchymal stem cells; ADPs: adipocytes; MDSCs: myeloid-derived suppressor cells, T cells; CAFs: cancer-associated fibroblasts; oCSCs: ovarian cancer stem cells; ECs: endothelial cells; M2: macrophages; EVs: extracellular vesicles.

Cellular communication among different cells in the tumor microenvironment is modulated by a variety of messages such as cytokines, growth factors, EVs, and miRNAs (Figure 1()), and how the microenvironment will interact is dependent on the needs of the CSC niche, and in what tumor process it will participate in. For example, a proinflammatory microenvironment, considered one of the hallmarks of cancer, is normally associated with tumor progression inducing proliferation, angiogenesis, and migration of cancer cells [28, 88]. IL-6 and CCL5 in the niche of the ovarian CSCs promote these processes, and CCL5 induces the differentiation of a subset of CSCs to generate ECs and support angiogenesis [54, 56, 59]. Other components of the proinflammatory network, such as IL-17 and IL-23, participate in the maintenance of the CSC niche promoting self-renewal, indicating their possible role in tumor initiation [41, 55].

Although IL-17 is secreted by CD4+ T cells and CD68+ macrophages in ovarian cancer, in other types of cancer, a population of FoxP3+ regulatory T cells (Treg), that under certain conditions express IL-17, plays a critical role in the regulation of CSCs [89]. Therefore, Treg could not only be modulating the tumor immunity by the inhibition of effector T cells but could also be regulating the tumor microenvironment and the release of different factors by the CSC niche.

Even though growth factors are considered one of the major regulators of the tumor progression process [60], they also participate in the self-renewal of CSCs and regulate their tumor initiation capacity [64, 71, 74]. This dual effect can be attributed to the heterogeneity of the ovarian tumor [90, 91]. Such a heterogeneity is also present in the ovarian CSC population [74, 92, 93] and could explain why the activation of the NF*κ*B-STAT3 signaling in one subset of CSCs (CD133+) induces self-renewal while in another subset (CD44+) it induces differentiation to ECs [55, 59]. The presence of a different CSC population could also explain why different factors contribute to CSC self-renewal, though this could be attributed to the activation of the same signaling pathway by different factors as well.

The role of microRNAs and EVs in the interaction between ovarian CSC niche and the tumor stroma is still an area of ongoing investigation, but its importance in gene regulation and cell communication supports the idea that they must play an important role in the self-renewal of ovarian CSCs.

Finally, it is worth mentioning that the microenvironment of the fallopian tube epithelium (FTE) could be a contributing factor to the CSC niche, given that there are several hypotheses that this is the site where ovarian cancer originates [94]. The identification of a stem cell niche in the FTE and the presence of a cancer-prone stem cell niche in the mesothelium and tubal (oviductal) epithelium support the idea that the FTE could play a role in the maintenance of the CSC niche [95, 96].

Understanding these interactions and what is the contribution of the ovarian CSC niche and of the other components of the tumor in the development of ovarian cancer will allow us to gain the knowledge needed to generate therapies against tumor progression and relapse.
